# A Markov Switching Approach in Assessing Oil Price and Stock Market Nexus in the Last Decade: The Impact of the COVID-19 Pandemic

**DOI:** 10.1177/21582440231153855

**Published:** 2023-02-20

**Authors:** Seuk Wai Phoong, Masnun Al Mahi, Seuk Yen Phoong

**Affiliations:** 1 Universiti Malaya, Kuala Lumpur, Malaysia; 2 BRAC University, Bangladesh; 3 Universiti Pendidikan Sultan Idris, Tanjung Malim, Malaysia

**Keywords:** oil price, stock price, breakpoint unit root test, Markov switching model, COVID-19

## Abstract

We revisit the oil price and stock market nexus by considering the impact of major economic shocks in the post-global financial crisis (GFC) scenario. Our breakpoint unit root test and Markov switching regression (MRS) analyses using West Texas Intermediate (WTI) oil price and Standard & Poor’s 500 (S&P 500) market index show that among the major economic events, the recent coronavirus (COVID-19) pandemic is the most significant contributor to market volatilities. Furthermore, our MRS results show that the relationship between oil price and the stock market is regime-dependent; the stock market experiences substantial and positive shocks in a volatile oil price regime. Our results provide valuable insights to investors and policymakers regarding risk management and financial market stability during economic crisis periods, specifically during the COVID-19 pandemic.

## Introduction

Stock markets are often linked to economic performance, and oil price is one of the commonly accepted economic phenomena that affect stock returns (Tchatoka et al., 2019). The relationship between oil prices and the stock market has been the subject of interest, especially today due to the dynamic nature of the oil prices and stock market nexus. Interest in the stock market movement re-emerged after the previous financial crisis of 2008 to 2009. Subsequent events, such as the ongoing European debt crisis, the oil price collapse in 2014, the United Kingdom’s Brexit referendum in 2016, and the United States (US) decision to leave the Paris Climate agreements are among the major global events during the last decade (Antonakakis et al., 2013; Bachmann et al., 2013; Baum et al., 2010; Bloom, 2009; Caggiano et al., 2017).

Recently, the oil and stock markets are crisis-ridden due mainly to the breakdown in negotiations between the Organization of the Petroleum Exporting Countries (OPEC) and non-OPEC members led by Russia and the outbreak of the Coronavirus (COVID-19) pandemic. Although the COVID-19 risk is somewhat transmitted to economic activities (Hasan, Mahi, Sarker, & Amin, 2021); the oil market appears to have been the primary receiver of volatility spillovers along with the financial markets due to the dramatic collapse of oil prices during the pandemic (Arafaoui & Yousaf, 2022; Yousaf, 2021). Also, Goldman Sachs highlighted the rapid drop in global storage availability and disruptions in physical distribution networks in the current scenario, placing continued pressure on prices (Masson & Winter, 2020).

The rapid spread of the pandemic adversely impacted the global financial markets and created an unprecedented level of risk, irrespective of the type of stock market (Hasan, Mahi, Hassan, & Bhuiyan, 2021), causing investors to suffer significant losses in a brief period (Zhang et al., 2020). The EU and US equity markets dropped by as much as 30% (Gormsen & Koijen, 2020). Market volatility has had a significant positive link with the new infection and volatility (Albulescu, 2021). Notably, the announcements of government social distancing measures had a direct negative effect on stock market returns due to their adverse impact on economic activities (Nadeem, 2020). Baker et al. (2020) quantifies the impact of news related to COVID-19 on the stock market and concluded that it has had a much more significant impact on the market than other similar diseases, such as Ebola. The economic and financial implications of the COVID-19 pandemic are so substantial, with some researchers comparing it to the global financial crisis of 2008 (Ozili & Arun, 2020; Sharif et al., 2020). The pandemic also weakened the transmission of monetary policy to financial markets to a more significant degree (Wei & Han, 2021), making policy intervention ineffective for a substantial period.

The interconnectivity of the global economy and financial render both susceptible to crises, as the latter affects international investors’ decisions on asset allocation (Liu et al., 2020). In such market dynamics, one particular stock market that plays a pivotal role is the US stock market. Previous studies evidenced that the US stock market had a strong contagion effect during the earlier crises, particularly during the 2007 to 2009 global financial crisis (GFC), which is regarded as the first truly major global crisis since the Great Depression of 1929 to 1932 (Bekaert et al., 2014; Jin & An, 2016). During the crisis, the US stock market plummeted by 43%, the emerging markets by 50%, and the frontier markets by 60% (Samarakoon, 2011). Hence, the US stock market’s co-movement with oil price fluctuations implies special significance for the global economy and financial market in other crises, including the COVID-19 pandemic.

Due to the lack of scholarly endeavor to comprehend the impact of the COVID-19 pandemic with a broader time horizon, this paper assesses the relationship between oil prices and stock markets in the post-GFC period to understand the impacts of different events in the market dynamics, notably including the COVID-19 crisis period. The study enables us to capture a unique aspect that existing literature has yet to address—it compares the shocks generated in the market due to the COVID-19 pandemic and other events (i.e., the European debt crisis, Brexit). Previous studies focused on studying the impacts of COVID-19 mainly restrict their analysis within the pandemic period, thus failing to clarify the differential impact of the COVID-19 pandemic compared to other crises. Accordingly, we can concurrently observe the effects of adverse changes in oil prices, variations in market conditions over time, and the pandemic’s intensity (Ren et al., 2022).

To this end, we first aim to identify the structural changes in the oil price and stock market index using a breakpoint unit root test to understand the relationship. The recent economic crisis induced by COVID-19 has caused significant market fluctuations, thereby recognizing a structural break in the time series. Using the Chow test, we further confirm the structural break date (4/27/2020). After securing the series structural break, we investigate the oil-stock relationship using the MRS model. The MRS model results show that the oil-stock relationship is state-dependent. Notably, the association is significant in the “high oil price fluctuation” state—an unstable oil market creates a positive shock in the stock market returns.

Accordingly, our study makes a three-fold contribution. First, we contribute to the existing literature by providing a comparative impact of the COVID-19 pandemic on the oil-stock dynamics and offer the latest empirical insights. As the COVID-19 pandemic has a time trend, the interactions between various economic variables entailed in this process would change over time (Wen et al., 2022). Second, from the methodological aspect, we offer empirical evidence using a nonparametric approach, while most of the earlier studies used parametric models to examine oil market shock and stock market volatilities (Kilian & Park, 2009; Lv et al., 2020; Mollick & Assefa, 2013; Phan et al., 2015; Ready, 2018). However, one fundamental limitation of the studies employing parametric models is that these models may not uncover the underlying relationship and how it has changed over time. Notably, due to many (known and unknown) events that may have had significant impacts on the oil-stock price relationship, the parametric model appears too restrictive for capturing the nature, and the extent, of changes in the underlying relationship (Silvapulle et al., 2017). Finally, our analysis of factors in the market dynamics during the COVID-19 pandemic and other global politico-economic phenomena has significantly impacted oil-stock relationships in the post-GFC scenario. Accordingly, our study offers the prospect of contrasting the recent COVID-19 and the plummeting oil price-induced market turmoil with other noteworthy events disrupting the market equilibrium. Hence, the findings are essential for policymakers on the onset of ongoing critical economic conditions and the volatile oil market condition induced by the COVID-19 pandemic and oil price war.

The remaining part of this article is organized into several sections: Section 2 discusses the methodological aspects of the study. Section 3 presents the relevant findings and discussion. Finally, Section 4 summarizes the results and highlights the conclusions of our research.

## Oil Price and Stock Market Nexus

Oil is considered a strategic commodity for economies as oil price fluctuations is not only limited to petroleum or other commodity markets but notably affect the financial markets, particularly stock markets (Mensi et al., 2022). Mensi et al. (2022) highlighted the oil-stock market linkages from two specific channels—microeconomic and macroeconomic. From the microeconomic theoretical perspective, cash flow and discount rate are two crucial factors that affect stock values in the market. Generally, a steeper oil price is linked with a higher rate of inflation in the economy, which leads to a higher interest rate, thus resulting in a higher discounting factor in valuing stocks (Basher & Sadorsky, 2006). Consequently, from the second or macroeconomic theoretical channel, oil price fluctuations are supposed to induce central banks to adjust interest rates to control inflation, leading to a decrease in stock prices (Basher & Sadorsky, 2006; Mensi et al., 2022).

However, there has been little agreement regarding the effects of oil price changes on stock performances, as the relationships are not straightforward. Mainly, the heterogeneity of the impact of oil price on stock prices, owing to the nature of the business, that is, oil-producing or oil-consuming company (Gomes & Chaibi, 2014; Kumar et al., 2012; Lv et al., 2020), as well as the oil status of the economy, that is, oil-exporting or oil-importing economy (Filis et al., 2011; Salisu & Isah, 2017; Tchatoka et al., 2019) as highlighted in existing studies.

The oil price effects are different for the markets in countries that are oil exporters compared to those that are oil importers (Degiannakis et al., 2018; Guesmi & Fattoum, 2014; Salisu & Isah, 2017). An oil price increase will likely affect an oil-exporting country positively, as their income will increase. The income increase is expected to result in a rise in expenditure and investments, which creates greater productivity and lower unemployment. Therefore, stock markets tend to respond positively to such an event (Arouri & Rault, 2012; Park & Ratti, 2008; Wang et al., 2013). On the contrary, an oil price increase for an oil-importing country will lead to higher production costs, as oil is one of the most critical production factors (Arouri & Nguyen, 2010). It is transferred to the consumers, leading to lower demand and, thus, consumer spending (Hamilton, 1996) and lower production (Lardic & Mignon, 2006). In such a case, stock markets would react negatively (Filis et al., 2011; Sadorsky, 1999).

### Earlier Empirical Studies

The US remained a net crude oil importer until 2020 (EIA, 2022). Previous studies used different methodologies to examine the relationship between oil prices and stock market in the US. One of the leading studies investigating the impact of oil price on the US stock market is that of Kilian and Park (2009), who considered the crude oil price as an exogenous shock in their structural VAR model, and reported that the shocks differ substantially, depending on the underlying causes of the oil price increase—whether the change in the price of oil is driven by demand or supply shocks in the oil markets. However, irrespective of the sources, their study estimated that the demand and supply shocks in the global crude oil market jointly account for 22% of the long-run variation in the US’ real stock returns. Their findings confirm the strong impact of oil price shocks on the US stock market. Tsai (2015) examines the US stock returns due to oil price shocks in the pre-crisis, during the financial crisis, and post-crisis scenarios using firm-level stock returns. By estimating OLS with panel-corrected standard errors, the author finds that stock returns to an oil price shock for most sectors within the crisis period are generally positive and heterogeneous, thus concluding that the crisis period and structural break have substantial impacts on the oil-stock price nexus.

An earlier study by Sadorsky (2008) used the generalized least squares (GLS) corrects for autocorrelation and heteroskedasticity in panel data sets to assess the impact of oil prices on firms of different sizes listed in the S&P 1500 and find a significant relationship between oil price movements and stock prices to varying extents for firms of various sizes. Many studies have also investigated the oil-stock price nexus and used GARCH models to examine market volatilities. Several examples of such studies are based on the US stock market employed different versions of GARCH models, including GARCH (1,1) (Falzon & Castillo, 2013; Phan et al., 2015), MGARCH-DCC (Mollick & Assefa, 2013), BEKK-GARCH (Lv et al., 2020) as well as MRS-GARCH (Ready, 2018).

Other studies used different methodological stances to investigate the relationship more meticulously. These studies mainly considered the asymmetry in the relationship. For example, Sim and Zhou (2015) examine the relationship using the quantile-on-quantile approach for oil prices and the US stock market. They reported two key findings—large and negative oil price shocks (i.e., low oil price shock quantiles) affect US equities positively when the US market is performing well (i.e., at the high return quantiles); while negative oil price shocks impact the US stock market, the influence of positive oil price shocks is weak. Bašta and Molnár (2018) analyzed the different time frequencies using wavelet transformation and used the implied and realized volatilities in the US market. They reported different results for both types of volatilities considered—implied volatility of the stock market leads to the implied volatility of the oil market. However, no such relationship was observed for realized volatilities.

Bahmani-Oskooee et al. (2019) utilized an asymmetric Granger causality test and failed to find a significant long-run causal relationship between oil prices and stock returns of nine different sectors of the US economy. Alternatively, Bu et al. (2020) employed the copula-MIDAS-X model to capture low-frequency to high-frequency data to examine the relationship between oil prices and the US stock market (S&P 500 index). Their results show that the relationship is asymmetric, and the dependence on oil and stock markets is influenced by aggregate demand and stock-specific negative news.

Although studies started considering the nonlinear or asymmetric aspects of the oil-stock nexus, specifically in the US market, they lack empirical evidence on how the recent significant events played a role in the relationship. In particular, existing studies failed to accommodate the impacts of structural break (or breaks) in the relationship and its potential influence in describing the asymmetry. Hence, the literature lacks a comprehensive interpretation that gains critical momentum in light of the sudden shock brought about by the COVID-19 pandemic.

## Materials and Methods

This section explains the empirical approach taken to investigate the oil-stock nexus. We first describe the empirical approach to identify the potential structural breaks due to different significant events over the last decade that we focus on for this study. Then, we explain the methodological aspects of the MRS model. We subsequently present the data and variables of the study.

### Breakpoint Unit Root Test

The empirical analysis in a time series usually begins with investigating the variables’ order of integration by applying the unit root tests. However, time series components, including seasonality components, trends, cyclical, and irregular changes (Mahi et al., 2020), and sudden economic or financial market shocks can create structural break(s) in the series. When there is a structural break(s) in the time series, the conventional unit root tests’ power is unstable (Sun et al., 2017). Moreover, Perron (1989) argues that breakpoints and unit roots are related, and conventional unit root tests are biased when determining the correct order of integration. Therefore, this study utilizes the Perron (1989) breakpoint unit root test method to determine the presence of unit roots and accommodate the structural break(s) in the time series of the variables under consideration. To determine the break in the intercept of the time series variable, the following general specification can be used;

(1) $$DU_{t}(T_{b})=1\;(t\geq T_{b})$$

where 1(·) indicates the value 1 if the argument (·) is true (or when there is a break) or 0 otherwise.

Accordingly, we specify the Dickey-Fuller regression to identify unit-root in the time series with intercept break as follows:

(2) $$y_{t}=\;\mu +\beta_{t}+\theta DU_{t}\left(T_{b}\right)+\alpha y_{t-1}+\sum_{i=1}^{k}c_{i}\Delta y_{t-1}+\mu_{t}$$

The model yields a test of a random walk against a stationary model with an intercept break. Also, as with the conventional Dickey-Fuller unit test, to eliminate the error correlation structure’s effect on the asymptotic distribution, the *k*-lagged differences of *y* are included in the equation. Also, we consider the Innovational outlier model that assumes the break takes place gradually, with a break following the same dynamic path as the innovation.

### Markov Switching Model

Regression analysis is a standard tool for exploring the correlations between continuous variables. There are three main types of multiple regression: simultaneous regression, hierarchical regression, and stepwise regression, to examine the association of the variables and to predict a particular outcome. However, the time series components such as linear trends, irregular patterns, and seasonal changes affect the findings’ results, which lack precision and accuracy (Phoong et al., 2019). Accordingly, the MRS framework is advantageous as the dataset incorporates several economic, financial, and geopolitical events (as outlined earlier) relevant to the oil and stock market dynamics.

Considering the analytical value of nonlinearity in the oil-stock nexus, we investigated the relationship using the Markov-switching regression (MRS) model. This technique is advantageous compared to conventional linear regression as the nonlinear nature of the time series might result in findings that lack precision and accuracy (Phoong et al., 2020). A switching regression helps us detect the existence of nonlinearity in the relationship. The MRS model can capture asymmetry or nonlinearity in economic/financial time series relationships. This approach allows model parameters to switch between different regimes, while other regime-dependent parameters can be estimated (Uddin et al., 2018). Also, the MRS framework has been proven useful when the adjustment seems to be mainly driven by exogenous events (Basher et al., 2016). Therefore, the technique helps detect whether and how the oil market fluctuations affect the return in the stock market.

The regression model without switching is:

(3) $$y_{t}=\alpha x_{t}+\varepsilon_{1},\varepsilon_{1}\;\sim i.i.d.\;N(0,\sigma^{2}),$$

The *x* is a 1 × *m* exogenous variables and coefficient of the independent variables. The evolution of the variable $s_{t}$ may be dependent upon $s_{t-1,\;}s_{t-2},\ldots ,\;s_{t-n}$ and hence the process of the discrete variable, $s_{t}$, is named as *n-*th order Markov switching process.

The first-order Markov switching process with the following transition probabilities:

(4) $$P\left(S_{t}=1|S_{t-1}=1\right)=p=\frac{\exp(p_{0})}{1+\exp(p_{0})}$$

(5) $$P\left(S_{t}=0|S_{t-1}=0\right)=q=\frac{\exp(q_{0})}{1+\exp(q_{0})}$$

where $p_{0}$ and $q_{0}$ are unconstrained parameters. The transition probabilities for a two-state Markov switching are then iterated to obtain $P[S_{t}=jS_{t-1}=i$, *i, j* = 0, 1 (Kim & Nelson, 2007; Phoong et al., 2020). The transition probability estimation is essential as it provides information about each state’s expected duration of the switching model (or economic condition), providing information on the asymmetric properties of the business cycle.

Generally, a Markov switching regression model used in this study assumes that there is a different regression model correlative with each regime. The unobservable variable, $X_{t}$ and $R_{t}$, the conditional mean of $y_{t}$ in regime *m* (*m* = 1, 2) for a two-regime model can be written as:

(6) $$y_{t}\left(m\right)=\propto_{m}X_{t}^{\prime }+R_{t}^{\prime }\beta$$

where $\propto_{m}$ and β are vectors of coefficients.

In this study, we consider the dynamics of West Texas Intermediate (WTI) and Standard & Poor’s 500 (S&P500) as state-dependent (the variable descriptions are provided in section 3.3). The parameters’ coefficients may differ for each state since the state can have low or high volatility, recession, or expansion (Phoong et al., 2020). The framework for the MRS is to be memoryless in each state (de Martino, 2018); the switching properties can be calculated using the following equation:

(7) $$\mathrm{lnS}\&P500_{t}=\mu_{i}+\beta \mathrm{lnWT}I_{t}+\varepsilon_{t}$$

where μ_*i*_= μ_1_ if *i* = 1 (or state 1), and μ_*i*_= μ_2_ if *i* = 2 (or state 2). Transition probabilities for a two-state model are:

(8) $$\left[\begin{array}{cc} p_{11} & p_{12}\\ p_{21} & p_{22} \end{array}\right]$$

where *p*_11_ + *p*_12_ = 1 and *p*_21_ + *p*_22_ = 1. The transition probability estimation is important as it provides information about each state’s expected duration of the switching model (or economic condition), providing information on the asymmetric properties of the business cycle.

We can obtain each regime’s average duration from the transition probability *p_jj_* (*j* = 1, 2). Precisely, the average duration of regime *j* (*j* = 1, 2) is specified as:

(9) $$D_{jj}=1/(1-p_{jj})$$

where *j* represents the state or regime.

### Data and Variables

We use daily WTI crude oil price and S&P 500 stock market index data to proxy the oil price and stock price, respectively. The WTI oil price is one of the most widely recognized international benchmarks for crude oil pricing. On the other hand, the S&P 500 is commonly regarded as the best single gauge of large-cap equities in the US, including 500 leading companies, and covers ~80% of available market capitalization. The data cover the period from January 1, 2010, to December 31, 2021. There is a total of 3,118 data points. Both data series are obtained from the Refinitiv Datastream database (Table 1).

**Table 1.** Descriptive Statistics.

Variable	*M*	*SD*	Minimum	Maximum
WTI	69.35371	22.41374	−37.63	113.93
S&P500	2252.16	883.6145	1,022.58	4,793.06
WTI return	0.000214	0.026740	−0.388293	0.300229
S&P500 return	0.000482	0.010661	−0.127652	0.089683

**Figure 1.**
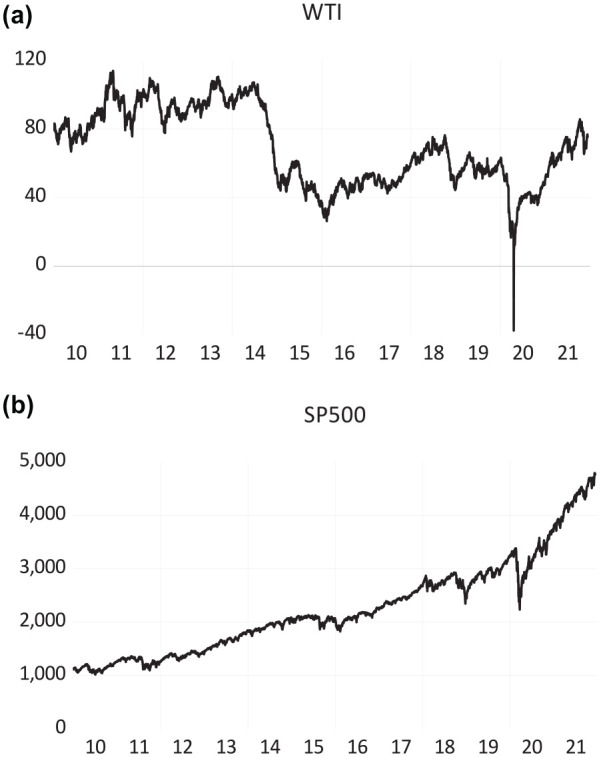
Variables plot: (a) time series graph of WTI and (b) time series graph of S&P 500.

Figure 1 shows the plot of the evolution of the daily prices of the WTI and S&P500 data series. The standard deviation for the oil price is high, as is the standard deviation for the S&P500. The larger the standard deviation, the more significant the change in the time series. We noticed that our post-GFC sample shows some fluctuations in the last decade. As mentioned above, the oil price collapse since 2014, the Brexit vote in the 2016 UK referendum, and the US decision to leave the Paris Climate Agreement are among the major global events during this decade. However, the oil and stock market suffered a massive shock recently, particularly in early 2020. The sharp spikes in both data series during the same period indicate that the oil-stock market nexus could suffer from a significant structural break; hence, the relationship needs a proper empirical revisit. Therefore, in the following section (Section 4), we investigate the relationship from alternative perspectives with relevant econometric techniques described subsequently.

For the empirical analysis, following Jiang et al. (2014), we calculated the log returns of the time series data by determining the difference between the two consecutive values, as follows:

(10) $$r_{t}=\ln\left(\frac{p_{t}}{p_{t-1}}\right)$$

## Results and Discussion

### Test of Structural Break and Unit Root

To begin with, in the investigation to find the oil-equity nexus, we first run the unit root test considering the possible break in each series, respectively. The results are presented in Table 2.

**Table 2.** Breakpoint Unit Root Test at First Difference.

Variable	*t* Statistics	Break date	Prob.
D (WTI return)	−97.17838	4/27/2020	<.01
D (S&P 500 return)	−64.58253	2/04/2010	<.01

*Note*. Probabilities are calculated using Vogelsang (1993) asymptotic one-sided *p* values. Prob. = probability.

Based on Table 2, the suggested break date is 4/27/2020 and 2/04/2010 for the WTI return and S&P 500 return, respectively. However, considering the influential transformation of shock from the oil market to the stock markets, we selected 4/27/2020 as the break date and then employed the Chow test to investigate the impact of this break date on the regression between WTI and S&P500 returns.

The null hypothesis of the Chow test is no break in the considered period (i.e., 4/27/2020). The Chow test fundamentally examines whether a single regression line or two distinct regression lines fit the dataset (Chow, 1960). The results in Table 3 show that the test statistics are significant at a 5% significance level for all the variables, thus, confirming the structural break during the break date considered in the time series variables under consideration.

**Table 3.** Chow Breakpoint Test.

Test	Test statistics	*p* Value	
*F*-statistic	17.13554	Prob. *F*(2,3112)	.0000
Log likelihood ratio	34.12755	Prob. Chi-Square (2)	.0000
Wald statistic	34.27107	Prob. Chi-Square (2)	.0000

**Figure 2.**
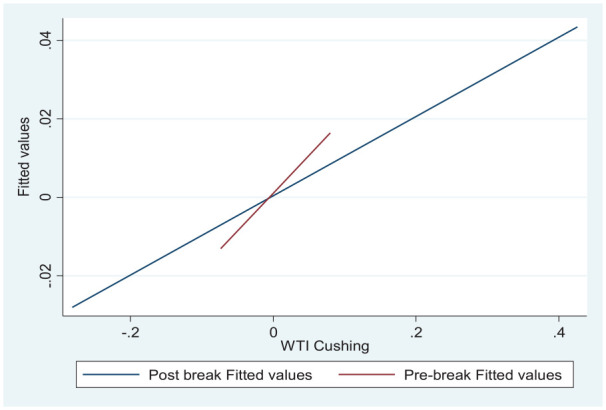
Fitted values graph for linear regression for a break date.

Therefore, the time series regression without considering the specified break date would result in a biased estimation. Figure 2 illustrates the considerable changes in the linear regression slope before and after the break date, which further justifies the specified break date.

Accordingly, we estimated the dynamics between WTI and S&P500 using a two-state MRS model, and the results are discussed in the following section.

### Results From Markov Regime Switching Model

A Markov switching model can capture the changes in the time series with the presence of structural breaks or regime changes. We investigated the possibility of a nonlinear relationship between oil price and stock price using the MRS model. The results are summarized in Table 4. We consider two regimes of the MRS model suggested by previous studies (Brunner, 1992; Neftçi, 1984). For the MRS outputs, the magnitude of volatility is determined by the overall size of each regime’s standard deviation (σ). The higher (lower) coefficients’ standard deviations regime is the high (low) volatility regime.

**Table 4.** Markov Regime Switching Regression Outputs.

Method: Markov switching regression (BFGS/Marquardt steps)							
Variable	Coefficient	*SE*	*Z*-statistic	Prob.	LL.	DW statistic	Schwarz criterion
Regime 1					10111.12	2.21	−6.471737
WTI	.093949	0.006882	13.65203	.0000			
Regime 2							
WTI	.833152	0.049297	16.90060	.0000			

*Note*. The number of states: 2. Initial probabilities obtained from ergodic solution. Common standard errors and covariance using a numeric Hessian Random search: 25 starting values with 10 iterations using 1 standard deviation (rng = kn, seed = 798,178,604). Convergence was achieved after 18 iterations. *p* Value is reported in the parenthesis. *SE* = standard error; *SD* = standard deviation; LL = log-likelihood.

A Marquardt step is used in the Markov switching regression model to estimate an unobserved state’s parameters for the MRS estimation in Table 4. Accordingly, we define regime 1 as the “low oil price fluctuation” state and regime 2 as the “high oil price fluctuation” state. For regime 1, the impact of WTI on the S&P500 is significant at 5% significance. On the other hand, we find that the impact of WTI on the S&P 500 is positive and significant at 1% significance level for regime 2. This positive association in regime 2 indicates that a high price fluctuation in the oil market creates a positive stock market shock. In other words, the oil price shock transition is significant in the volatile market condition. Our findings align with previous studies that the oil-stock relationship is unstable and varies in different phases over time (Balcilar et al., 2015; Lee & Chiou, 2011).

We also noticed that the standard error of the regression coefficient in regime 2 (0.04929) is around seven times larger than the standard error of the coefficient in regime 1 (.006882). A standard error was used to measure the variability of the coefficient. The standard error of the coefficient is always positive. The smaller the standard error, the more precise the estimate. From the findings, although the standard error in regime 2 is higher than in regime 1, both values are close to 0. This indicates that the statistic has no random error, which also means that the sample of data is sufficient and the estimated value is close to the true value.

The range for Durbin Watson statistics is 0 to 4, where an acceptable range is 1.50 to 2.50. The value below 1.5 indicates the presence of positive autocorrelation, while the statistic above 2.5 indicates the presence of negative autocorrelation. The Durbin-Watson statistic is 2.21, which is within the acceptable range. This means that no first-order autocorrelation happens in this regression model.

Table 5 presents the probability of transition from one regime to another. The transition probabilities from regime 1 to regime 2 (*p*_12_) are lower than those from regime 2 to regime 1 (*p*_21_). This indicates that regime 1 is relatively permanent. The process of transition from regime 1 to regime 2 is very low. Moreover, the expected duration of regime 2 is close to 41 days, and the anticipated period of being in regime 2 is close to 4 days. This confirms that regime 1 is more stable than regime 2. Furthermore, *p*_11_ and *p*_22_ have a high value; thus, we reject the null hypothesis of no regime shifts.

**Table 5.** Transition Probabilities and Expected Durations.

Transition probabilities			
*P*_11_	*P*_12_	*P*_21_	*P*_22_
.975390	.024610	.273415	.726585
Expected duration			
	DU_1_	DU_2_	
	40.63425	3.657450	

*Note*. The transition probabilities are reported as *P_ij_*. The expected duration of being in state “*i*” is reported as DU_*i*_, that is, DU_1_ for state 1 and DU_2_ for state 2.

### Robustness Test

To check the robustness of the MRS analysis, we used an alternative proxy for stock market return. We used the Dow Jones Industrial Average (DJIA) index return and regress against the WTI return series. Unlike the S&P 500, DJIA represents 30 large-cap companies. The index is not weighted by market capitalization; rather, the index was calculated by summing the listed stock prices divided uniquely by a factor called “Dow divisor” to smooth over infrequent changes like stock splits and new index constituents (Langley, 2020). Hence, the choice of DJIA offers a distinctive prospect to investigate the stock market movement due to changes in oil prices or the shock generated in the oil market.

A Bai-Perron breakpoint test was used to investigate the potential structural change or breaks in the variables’ series. The results are presented in Table 6.

**Table 6.** Bai-Perron Test Outputs—Alternative Stock Market Index.

Break test	*F*-statistic	Scaled	Critical
		*F*-statistic	Value
0 versus 1*	17.96847	35.93694	11.47
1 versus 2	0.944493	1.888985	12.95

* The test is significant at the .05 level.

The Bai-Perron test is an algorithm for determining the structural break in a linear regression model by trimming 15% of the data. The purpose of trimming the 15% at the beginning or end of the sample data is to avoid the presence of serial correlation or heterogeneity in the data or errors that might occur across the segments. Based on the results in Table 6, there is 1 structural break (2/25/2020) in the model. Next, a two-state MRS regression was used to examine the correlation between oil price and stock price. The sample data ranged from 1/1/2010 until 12/31/2021. The duration of the sample data is similar to the S&P500. A total of 3,118 data was calculated using the MRS model, and the results are reported in Table 7.

**Table 7.** Markov Switching Regression Outputs—Alternative Stock Market Index.

Method: Markov switching regression (BFGS/Marquardt steps)							
Variable	Coefficient	*SD*	*Z*-statistic	Prob.	LL.	DW statistic	Schwarz criterion
Regime 1							
WTI	−.031070	0.006577	−4.723885	.0000	9980.80	2.21	−6.390142
Regime 2							
WTI	−.717868	0.043757	−16.40569	.0000			

*Note*. The number of states: 2. Initial probabilities obtained from ergodic solution. Common standard errors and covariance using a numeric Hessian Random search: 25 starting values with 10 iterations using 1 standard deviation (rng = kn, seed = 12,346,587,827). *p* Value is reported in the parenthesis. *SE* = standard error; *SD* = standard deviation; LL = log-likelihood.

The presence of two regimes is evident in Table 7. We defined regime 1 as the “high oil price fluctuation” state and regime 2 as the “low oil price fluctuation” state based on the standard deviation (SD) values of the regression coefficients, respectively. Similar to our main findings, our robust MSR confirms a significance on the stock market when the oil price fluctuations are high. Moreover, the relationship remains significant and negative in the low oil price fluctuation state, similar to earlier findings. Besides that, the Durban Watson statistic is 2.21, comparable with the findings in Table 4, indicating that no autocorrelation occurs in the regression model. Thus, the results confirm switching behavior in the oil-stock nexus and a positive shock transition in the stock market when oil price volatility is high in the market. Furthermore, the transition probabilities and expected duration results presented in Table 8 show comparable values. Regime 1 is more permanent than regime 2, as the anticipated duration for regime 1 is 232 days, and the expected time of being in regime 2 is 1 day. This confirms that regime 1 is more stable than regime 2, which agrees with the findings of the S&P500. Consequently, we demonstrated that the oil-stock relationship is subject to rapid regime changes due to price shocks.

**Table 8.** Transition Probabilities and Expected Duration—Alternative Stock Market Index.

Transition probabilities			
P_11_	P_12_	P_21_	P_22_
.995692	.004308	.999829	.000171
Expected duration			
	DU_1_	DU_2_	
	232.1248	1.000171	

## Conclusions

The relationship between oil price and stock price is critical, and the significance of this study stems from the economic and policy-related significance of the oil-stock nexus. The relationship dynamics are also subject to change over time from sudden changes in the economy, financial market, or oil market, and this study aims to revisit this relationship against the backdrop of several global events in the post-GFC scenario. The recent COVID-19 pandemic crisis has created a substantial shock in the economy and markets, and to analyze the effect of the pandemic on the relationship, we used the daily data of WTI crude oil price and S&P 500 stock market index data to proxy the oil and stock prices.

We consider the possibility of a structural break in the time series and employed the “breakpoint unit root test.” We uncovered evidence of a significant structural break on 7/28/2020 and confirmed it using the Chow breakpoint test. We also examined the oil-stock relationship, considering the nonlinearity in the time series. We employ a two-state MRS model and discovered significant variations in the relationship between the two regimes. Notably, the relationship is significant in the “high oil price fluctuation” state, or stock market return is significantly affected by shocks generated in a volatile oil price regime. Hence, we empirically confirmed that the oil-stock relationship is nonlinear and asymmetric, and the stock market is susceptible to fluctuations when oil prices are unstable.

Our findings are of particular significance for investors and policymakers. First, policymakers can formulate appropriate strategies to keep oil prices stable, which will help prevent market contagion. Specifically, the US, the producer of WTI crude oil, is expected to prioritize dealing with risks induced by the pandemic. Second, our results suggested that the most significant break occurred during the COVID-19 period compared to other events, including the recent oil price war. Policymakers should also concentrate on alternative energy sources, such as renewable energy, to decrease the high dependence of economic activities on oil. Third, stock market investors can also monitor the changes in oil prices while investing and constructing portfolios. We suggest that achieving a well-diversified portfolio should involve the consideration of oil price shocks, which, as a consequence, could also help improve the accuracy of hedging against the risks generated by the high fluctuation of oil prices. Finally, the findings can be helpful to investors and financial market regulators—they need to be more vigilant to non-economic crises besides economic ones to adapt their investment strategies and minimize financial loss. Overall, these findings provide valuable insights to investors and policymakers on spillovers across non-economic (i.e., health) crises, oil price fluctuations, diversification, and risk management strategies in the stock market.

Our study is not without limitations, which can be addressed in future research with careful modeling and estimation. One such limitation is that we used only two variables; however, the oil-stock relationship can be affected by other factors as well. Future research can expand the model to a multivariate framework to include other financial or economic variables, such as the exchange rate, the stock market volatility index (VIX), the oil volatility index (OVX), and the like. Further studies with this method can be extended to a three-regime MRS model to measure the oil-stock nexus in three states: high oil price fluctuation, stable oil price condition, and low oil price fluctuation regime.
